# Supplementary material for: Robust analysis of allele-specific copy number alterations from scRNA-seq data with XClone
Source: Nat Commun. 2024 Aug 6;15:6684. doi: 10.1038/s41467-024-51026-0 (PMC11303794; doi:10.1038/s41467-024-51026-0)
Supplement: Supplementary file 3 — Description of Additional Supplementary Files [file 41467_2024_51026_MOESM3_ESM.pdf]

Description of Additional Supplementary Files

File Name: Supplementary Data 1-12

Description:

1. Tumor single cell transcriptomics dataset information and CNA profiles.
2. BCH869 CNA ground truth.
3. Number of consensus cells and genes of five methods for benchmarking on BCH869 scRNA-seq dataset.
4. Running time and memory usage of five methods on two scRNA-seq datasets.
5. Benchmarking of methods in CNA detection in single cell transcriptomics data.
6. SNPs within significant DEGs on chromosome 14 in the BCH869 dataset.
7. GBM 2R Clone1 CNA ground truth.
8. Number of consensus cells and genes of five methods for benchmarking on 2R Clone 1 in GBM snRNA-seq dataset.
9. GX109-T1c CNA ground truth called from matched scDNA-seq data.
10. CNA ground truth for the simulation of allelic copy loss in GX109-T1c data.
11. Cell type annotation for the simulation of allelic copy loss in GX109-T1c data.
12. Benchmarking Performance on simulated GX109-T1c datasets.
